# The SUbventral-Gland Regulator (SUGR-1) of nematode virulence

**DOI:** 10.1073/pnas.2415861122

**Published:** 2025-03-10

**Authors:** Clement Pellegrin, Anika Damm, Alexis L. Sperling, Beth Molloy, Dio S. Shin, Jonathan Long, Paul Brett, Tochukwu Chisom Iguh, Olaf P. Kranse, Andrea Díaz-Tendero Bravo, Sarah Jane Lynch, Beatrice Senatori, Paulo Vieira, Joffrey Mejias, Anil Kumar, Rick E. Masonbrink, Tom R. Maier, Thomas J. Baum, Sebastian Eves-van den Akker

**Affiliations:** ^a^The Crop Science Centre, Department of Plant Sciences, University of Cambridge, Cambridge CB2 3EA, United Kingdom; ^b^Department of Biochemistry and Metabolism, John Innes Centre, Norwich NR4 7UH, United Kingdom; ^c^Department of Agriculture—Agricultural Research Service, Mycology and Nematology Genetic Diversity and Biology Laboratory, Beltsville, MD 20705; ^d^Department of Plant Pathology, Entomology and Microbiology, Iowa State University, Ames, IA 50011; ^e^Genome Informatics Facility, Iowa State University, Ames, IA 50011

**Keywords:** food security, plant-parasitic nematodes, effectors, effector regulation, transcriptional regulation

## Abstract

Pathogens must precisely tailor their gene expression to cause infection. However, a signaling cascade from host signal to effector production has remained elusive for metazoan pathogens. Here, we show that plants contain molecular signals, termed effectostimulins, that activate the first identified regulator of plant-parasitic nematode effectors. SUGR-1 directly binds effector promoters, and is central to a transcriptional network that activates 58 effector genes. Importantly, we demonstrate that downregulation of *sugr-1* inhibits parasitism, underlining SUGR-1 signaling as a valuable target for crop protection and food security. This, in the wider context of nematodes as parasites of humans and other animals, has scope for potentially broader impact: Disrupting effector production could, in principle, be applied to any pathogen that secrets effectors.

## Significance

We show that effector deployment at the earliest stages of infection by plant-parasitic nematodes is defined by a feedforward signaling loop, centered on a transcriptional regulator, SUGR-1, and stimulated by plant-derived signals. In conjunction with parallel understanding in prokaryotic systems, we posit a generalizable framework that applies to all pathogens which secrete effectors.

In humans, other animals, and plants, pathogen/parasite secretory/excretory products (often termed effectors) manipulate the host to benefit the invader (1, 2). These effectors can be recognized by the host, allowing the immune system to restrict infection, leading to an evolutionary arms race between host and pathogen (3, 4). Effectors, and the host genes that they interact with, therefore, sit at the crux of engagement between kingdoms of life, defining disease or resistance.

Recent and rapid advances in effector biology have shaped our understanding of effector function and importance (5). Effectors play pivotal roles during host invasion (6), immune suppression (2, 7) as well as modulation of host physiology and development (8) sometimes even culminating in the formation of novel organs (9).

Plant-parasitic nematode effectors are primarily produced in two sets of gland cells: one dorsal and two subventral gland cells (10). The subventral gland cells mainly produce early-stage effectors involved in root penetration and migration (11–19), while the dorsal gland produces those involved in immunity suppression and developmental reprogramming (9).

As a result, various defense strategies aim to interfere with effectors, e.g. through recognition by resistance genes or by targeting effectors directly via RNAi (20, 21). However, blocking the action of individual effectors will likely not lead to durable control of a given pathogen because effectors are at the interface of host–pathogen interactions and subject to intense evolutionary pressure. This has resulted in higher than background fixation of mutations compared to other genes (22), and the localization of effectors in genomic regions associated with higher mutation rates (23). This high rate of evolution, coupled with functional redundancy (24) and overwhelming numbers [in some cases hundreds (25)], impacts the robustness and practicality of effectors as targets for pathogen control. Indeed, resistance achieved through targeting effectors has been swiftly overcome (26, 27). A solution may be to target effectors indirectly by blocking the unifying aspects that regulate host:parasite biology during infection (28).

Effector production is precisely regulated in time and space to infect the host (29–32). In this regard, all pathogens must recognize they are inside the host to effectively alter their physiology and gene expression. However, to the best of our knowledge, a signaling cascade from host cue to effector production has not been defined in a metazoan pathogen.

Here, we define such a signaling pathway in a cyst nematode—devastating root pathogens of global agricultural importance that can cause yield losses of up to 90% in cereals (33) and 80% in potatoes (34). We show that in the beet cyst nematode *Heterodera schachtii*, plant signals termed effectostimulins activate the first identified regulator of plant-parasitic nematode effectors: *sugr-1.* We propose a model where, in a positive feedback loop, increased effector production facilitates host invasion, which in turn releases more effectostimulins. Finally, we demonstrate that targeting this signaling cascade by downregulating *sugr-1* inhibits parasitism, and translate these findings to the SUGR-1 homolog in the soybean cyst nematode *Heterodera glycines.*

## Results

### Nematodes Exposed to Root Extract Are Primed for Infection.

**Fig. 1.**
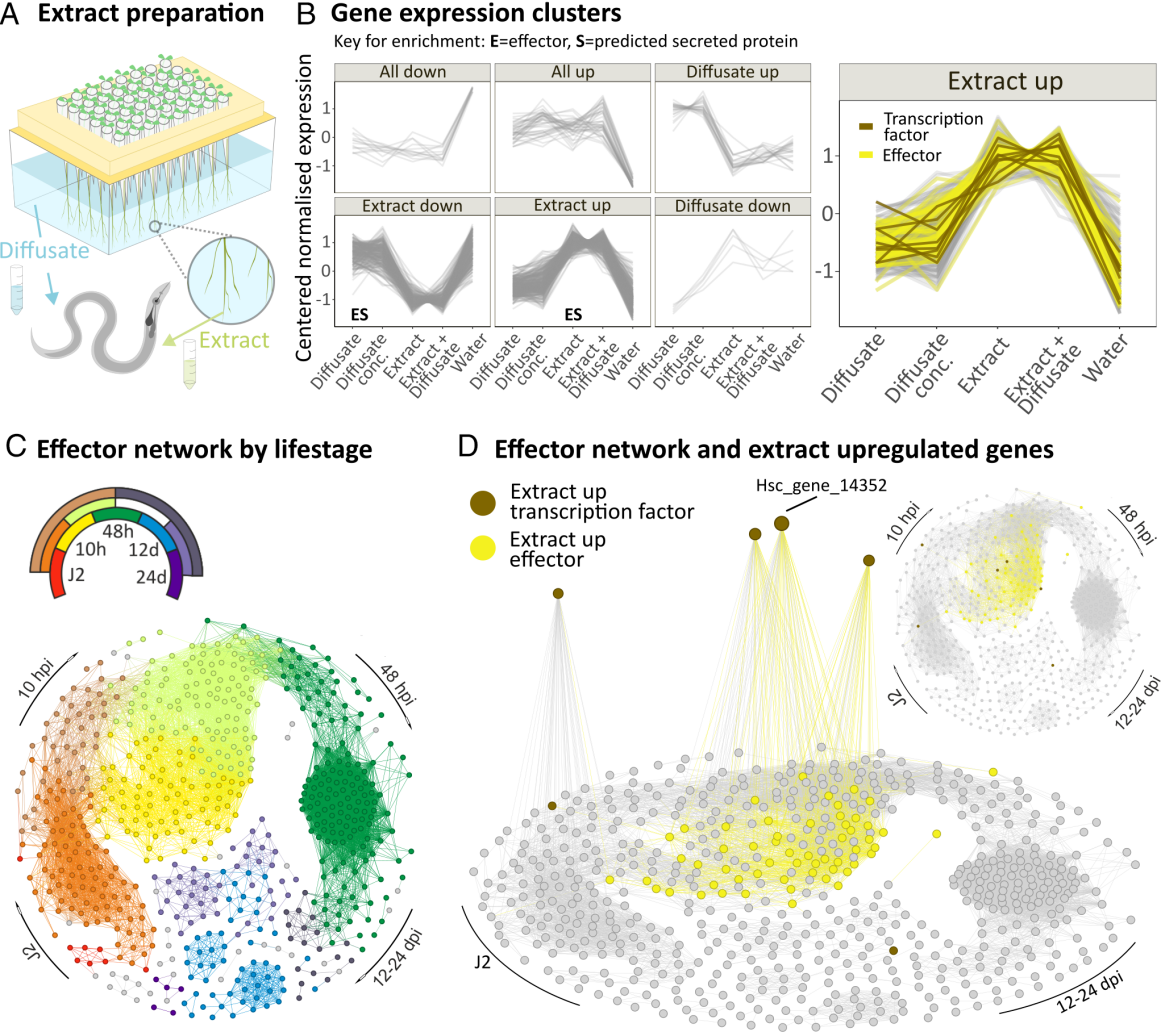
*H. schachtii* gene expression responds to host cues. (*A*) *S. alba* (white mustard) plants were grown in tip boxes filled with water. *H. schachtii* second-stage juveniles (J2s) were exposed to root diffusate (water) and/or extract prepared from the roots (n = 5). (*B*) Differential gene expression (n = 5; |log2FC| ≥ 0.5 & *P*adj ≤ 0.001) clusters that describe *H. schachtii* response to mustard root diffusate and extract. Enrichment was determined in hypergeometric tests (*P* < 0.01). (*C*) Transcriptional effector network, computed from independently generated expression data (31), where nodes represent effector genes predicted in ref. 25 and edges represent correlations in gene expression across the nematode life-cycle of 0.975 or above (distance correlation coefficient). Colors indicate the nematode life stages. (*D*) Transcriptional effector network highlighting effectors upregulated by mustard root extract in yellow. Extract upregulated transcription factors (TFs) with connections to the effector network (brown) are shown on the *z* axis where height is determined by connectedness to the effector network.

Most *H. schachtii* effectors are maximally expressed after the nematode has reached the plant (31). Therefore, we hypothesized that effectors, and indeed regulators thereof, might respond to plant signals. To simulate, and distinguish between, the perception of signals associated with host approach and host entry, we separated the molecules contained within roots (root extract), from those released into the rhizosphere [root diffusate (Fig. 1*A*)] of the host *Sinapis alba* (white mustard). Application of root extract and/or root diffusate altered the expression of 685 nematode genes, as evidenced by comparative RNAseq analysis, which were assigned to six distinct clusters (Fig. 1*B*). Extract application, whether in combination with diffusate or alone, had the dominant effect (88%, 602/685) on gene expression (*SI Appendix*, Fig. S1 and Fig. 1*B*). The “Extract up” cluster was highly enriched in predicted effectors, as defined in ref. 25 by homology and direct gland cell sequencing, and secreted proteins, as defined in ref. 25 by the presence of a signal peptide and absence of transmembrane or other targeting signals that would preclude secretion (hypergeometric test, 60/359 *P* = 3.1E^−30^, and 109/359, *P* = 2.4E^−27^, respectively).

The concerted upregulation of such a large number of sequence-unrelated effectors in response to root extract implies the existence of a master regulator. Based on the hypothesis that the expression of a positive regulator of effectors would correlate with effector gene expression, we investigated the eight TFs in the Extract up cluster. To prioritize investigation, we used independently generated expression data (31) from across the life cycle to compute distance correlation coefficients between these eight putative regulators and the entire predicted effectorome (25). Visualizing these distance correlation coefficients in a transcriptional network (Fig. 1*C*), highlights Hsc_gene_14352 as the most highly connected of the eight candidates, and indeed the second-most highly connected TF of any kind to the network (25). Hsc_gene_14352 is colocalized in the network with effectors expressed at the very earliest stages of host entry [measured 10 h post infection (hpi)]. Cross referencing the network with those effectors upregulated by root extract highlights this same time point (Fig. 1*D*), independently validating the observation.

### Identifying the SUbventral Gland Regulator 1 (SUGR-1).

**Fig. 2.**
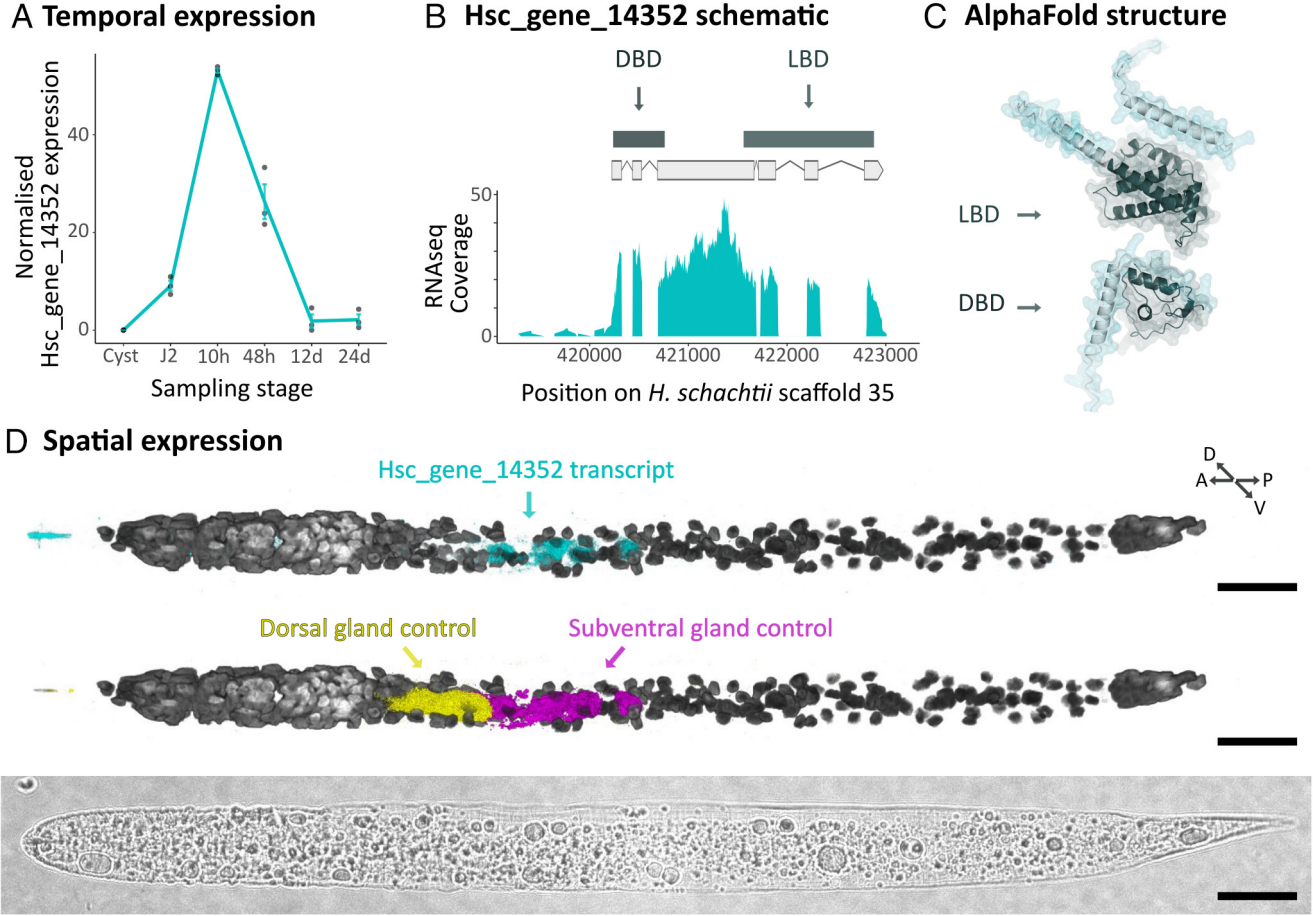
Characterization of Hsc_gene_14352. (*A*) Hsc_gene_14352 gene expression over the nematode life cycle. Data from ref. 31. (*B*) Hsc_gene_14352 gene model, predicted to encode both a C-terminal DBD and a predicted N-terminal LBD, and RNAseq coverage. (*C*) Hsc_gene_14352 alpha fold structure. (*D*) Multiplexed HCR in situ for Hsc_gene_14352 transcripts (*Upper Panel*, cyan), compared to dorsal gland (Hsc_gene_2729) and subventral gland (Hsc_gene_21727 *eng2*) control transcripts (*Middle Panel*, yellow, and magenta, respectively). Nuclei stained with DAPI are shown in gray scale. Brightfield is shown in the *Bottom Panel*. (Scale bars, 20 μm.)

Hsc_gene_14352 is a canonical nuclear hormone receptor, predicted to encode both a C-terminal DNA binding domain (DBD) and an N-terminal ligand binding domain (LBD), and is expressed principally at 10 h post infection (Fig. 2 *A*–*C*). Nuclear hormone receptors are known to regulate a variety of processes (e.g., response to developmental, environmental, and nutritional signals), and the family is expanded in nematodes (35). Nematode effectors are predominantly produced in two sets of gland cells: the two subventral gland cells being more active at early stages, while the dorsal gland cell becomes active at later stages of infection (36). Hsc_gene_14352 is reliably represented in targeted gland cell transcriptomic data (25) and we used Sperling prep. fluorescence in situ hybridization chain reaction (HCR) (37) to show that it is specifically expressed in the subventral gland cells (Fig. 2*D*). Taken together, these data show that Hsc_gene_14352 is expressed in the same cells, and at the same time, as subventral gland effectors.

**Fig. 3.**
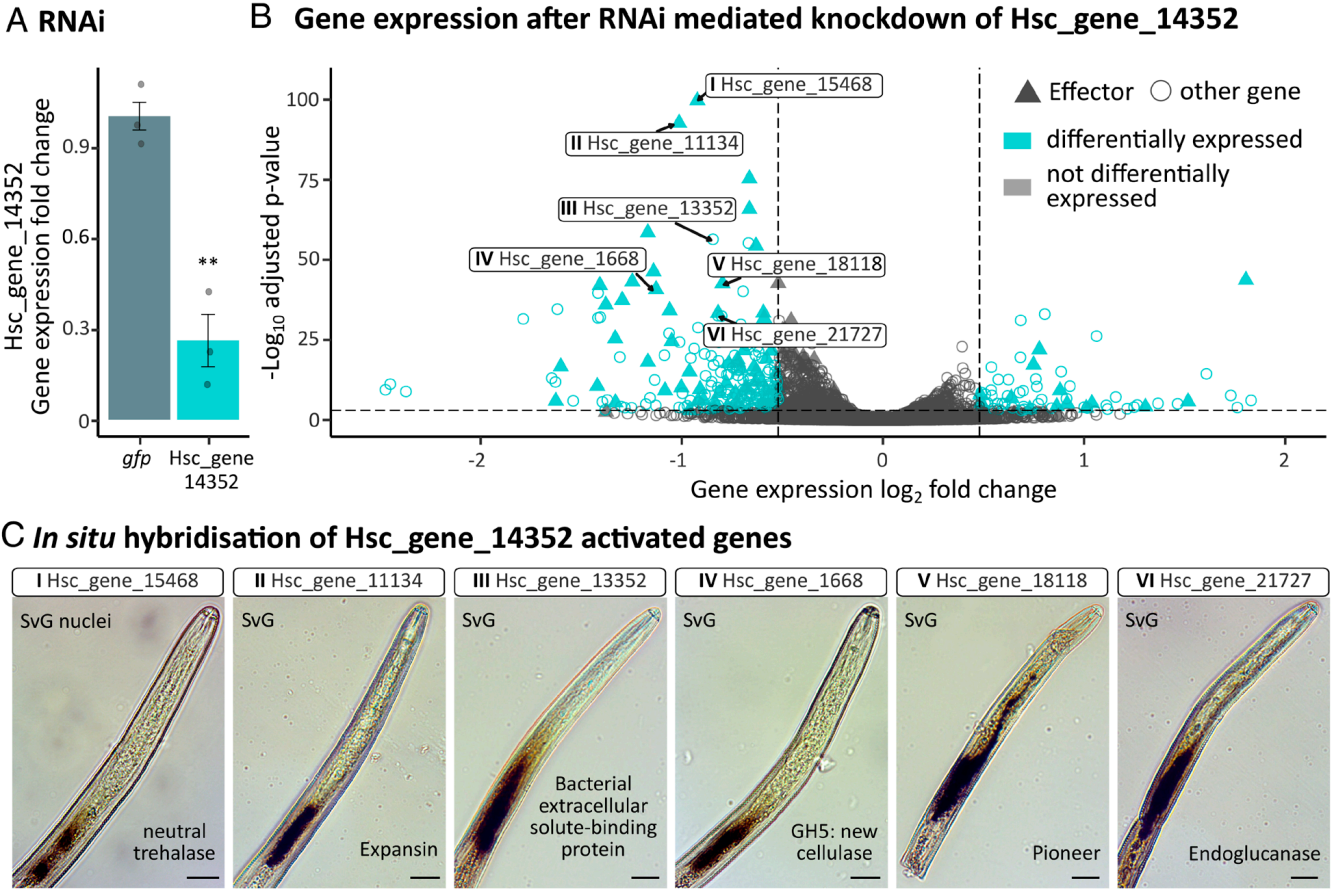
Hsc_gene_14352 is the SUGR-1. (*A*) Hsc_gene_14352 expression following RNAi-mediated knockdown compared to a *gfp* control. Gene expression was determined by qPCR and analyzed using the two-sample *t* test (**P < 0.01). (*B*) *H. schachtii* gene expression following Hsc_gene_14352 knockdown vs. *gfp* control. Differentially expressed genes (n = 3; |log2FC| ≥ 0.5, and *P*adj ≤ 0.001) are highlighted in cyan. Effectors (as predicted in ref. 25) are triangles. Six (roman numerals) selected Hsc_gene_14352-regulated effectors/effector candidates are indicated. Predicted secreted proteins are highlighted in *SI Appendix*, Fig. S2*A*. (*C*) In situ hybridization of six Hsc_gene_14352-regulated effectors/effector candidates. (Scale bars, 15 µm.)

Hsc_gene_14352 is predominantly a positive regulator of gene expression, as evidenced by comparative RNAseq analysis. There are 297 differentially regulated genes in Hsc_gene_14352-silenced J2s compared to control *gfp*-silenced J2s (n = 3, |log2FC| ≥ 0.5, and *P*adj ≤ 0.001), the vast majority of which (77%) are concordantly down-regulated with Hsc_gene_14352 (Fig. 3 *A* an *B*). The Hsc_gene_14352-regulon is enriched in GO terms associated with carbohydrate metabolic processes (GO:0005975), polysaccharide catabolic processes (GO:0000272), and the parent term cellulose metabolic processes (GO:0030245—Dataset S1 and *SI Appendix*, Fig. S2), consistent with functions of known subventral gland effectors in cell wall degradation and host entry (25). Indeed, Hsc_gene_14352 positively regulates 58 members of the predicted *H. schachtii* effectorome. Of those positively regulated effectors with experimental evidence of gland cell expression, 90% (18/20) are localized to the subventral gland, including several virulence determinants (11, 12, 14, 16, 17, 38). Interestingly, of the regulated genes encoding putatively secreted proteins, 63 (46.7%) are not known members of the *H. schachtii* effectorome, highlighting potentially novel effectors (*SI Appendix*, Fig. S2). Therefore, to validate a subset of six activated genes, including known and putative novel effectors, we confirmed subventral gland expression by in situ hybridization (Fig. 3*C*). These data unequivocally demonstrate that the expression of effectors in the subventral gland cells is regulated by Hsc_gene_14352, which we have named the SUbventral Gland Regulator 1 (SUGR-1).

### Effectostimulins, Small Heat-Stable Signals Inside Plant Roots, Trigger a Signaling Cascade in Nematoda That Upregulates Effectors.

**Fig. 4.**
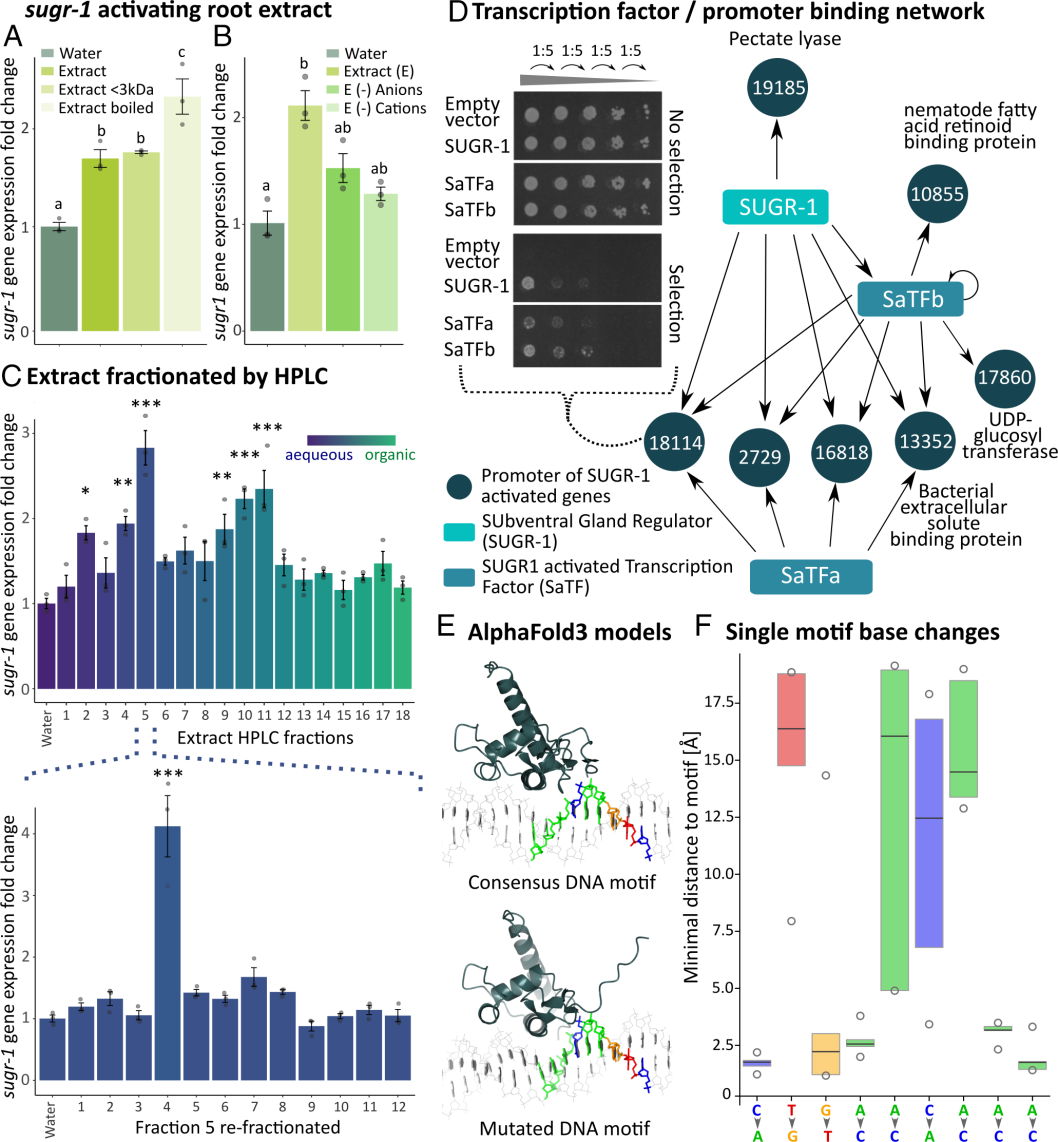
Signaling cascade regulating nematode effector production. (*A*) Effect of mustard root extract on *sugr-1* gene expression in *H. schachtii.* (*B*) Effect of ion depleted root extract (<3 kDa) on *sugr-1* expression. For *A* and *B*, treatments with the same letter are not statistically significantly different at *P* < 0.05 [Tukey HSD (*A*) or Dunn’s test (*B*)]. (*C*) Effect of mustard root extract (<3 kDa) fractions (fractionated by HPLC) on *sugr1* expression. Fraction 5 was refractionated (*Lower Panel*). Asterisks indicate treatments statistically significantly different to the water control (*P < 0.05; **P < 0.01; ***P < 0.001) as determined by Tukey HSD (Honestly Significant Difference) test. For *A*–*C* qPCR data were normalized using the Pfaffl method. (*D*) Network of TF:promoter interactions (Y1H data in *SI Appendix*, Figs. S4 and S5). Numbers indicate Hsc_gene name of corresponding gene. (*E*) Example SUGR-1 AlphaFold3 models predicted with a 29 bp Hsc_gene_21726 promoter region containing the SUG box version CTGAACAAA vs a mutated DNA motif (position 6; C→A). Only the SUGR-1 DBD is shown. (*F*) Predicted effect of DNA motif single base changes (A↔C or T↔G) on the minimal distance between SUGR-1 C-terminal loop and the DNA motif.

*sugr-1* is upregulated by root extract (Fig. 1*B*), and SUGR-1 in turn upregulates subventral gland effectors (Fig. 3*B*). To characterize the earliest parts of this signaling cascade in more detail, we sought to determine whether mustard root extract contains discrete activating signals. Removing molecules above 3 kDa from the extract, as well as heating the extract to 95 °C, did not reduce the activating effect (Fig. 4*A*, Tukey HSD, n = 3, *P* < 0.05), implicating at least one small heat-stable signal. Furthermore, extract (<3 kDa) depleted in either strong anions or strong cations could no longer significantly activate *sugr-1* transcription, perhaps indicating one or more charged signals (Fig. 4*B*, Dunn’s test, n = 3, *P* > 0.05). Finally, and importantly, separating the contents of root extract (<3 kDa) based on their solubility in water, using high-performance liquid chromatography (HPLC), revealed multiple fractions, grouped in three peaks, that significantly activate *sugr-1* gene expression (Fig. 4*C*, Tukey HSD, n = 3, *P* < 0.05). Upon additional fractionation of Fraction 5 (which contained the strongest peak) the signal could be further isolated (Fig. 4*C*). Taken together, these data are most easily explained by a minimum of three discrete small molecule signals found inside plant roots that activate *sugr-1* gene expression, and thereby effector expression. We termed this class of signals effectostimulins. To explore the concept of effectostimulins further, we expanded the data to different hosts and nonhosts. While root extract of the host plant *Arabidopsis thaliana* actives *sugr-1* gene expression to a similar degree as mustard, no activation could be observed for the nonhosts tomato and rice (*SI Appendix*, Fig. S3).

To characterize the later parts of the SUGR-1 signaling cascade in more detail, we focused on the finding that SUGR-1 also controls the expression of three TFs: two SUGR-activated TFs (SaTF a and b) and one SUGR-repressed TF (SrTF). Yeast-one-hybrid screens showed that SUGR-1, SaTFa, and SaTFb all directly bind effector promoters in yeast in a partially overlapping manner, such that seven of the eleven tested effector promoter regions are bound by at least one TF, and four are bound by all three. Interestingly, SUGR-1 also directly binds the promoter region of SaTFb in yeast (Fig. 4*D* and *SI Appendix*, Figs. S4 and S5). These TFs may, therefore, have the capacity to directly regulate expression of these genes in *cis*.

SUGR-1-regulated interactions in *cis* are likely mediated by a conserved DNA motif (*SI Appendix*, Fig. S6*A*). A differential motif discovery algorithm identified a homologous sequence enriched in the promoters of SUGR-1-regulated effectors, subventral gland effectors, and early-stage effectors from the J2 to 10 h post infection supercluster (as defined in ref. 31). Comparison between the enriched motifs reveals a conserved “core” of TG[C | A]AC, which is also the reverse complement of a canonical nuclear hormone receptor binding site (39, 40). We term this motif the SUbventral Gland box (SUG box) following the established convention (22).

We predicted putative SUGR-1:DNA complex formation using AlphaFold3 and a 29 bp promoter region of the cellulase effector Hsc_gene_21726, centered on the SUG box version CTGAACAAA. As expected, SUGR-1 was predicted to interact with the cognate DNA motif and, importantly, mutating a single nucleic acid within the motif (position 6; C→A) resulted in a loop of the first 10 amino acids at the SUGR-1 C-terminus dissociating from the DNA (Fig. 4*E*). Single base-pair changes (A↔C or T↔G) scanning across the motif produced similar dissociations for positions, 2, 5, and 7 (Fig. 4*F* and *SI Appendix*, Fig. S6*B*). Given the consistency in this finding, the distance between the C-terminal loop of SUGR-1 and the DNA motif was used as a proxy to compare SUGR-1:DNA interactions of 14 further promoter regions (total of 15), each containing CTGAACAA[A|T], with 15 random promoter regions in randomly selected promoters. For motif-containing promoter regions, 85.3% of the models predicted a minimal distance below 4.5 Å between the DNA motif and the C-terminal loop (cutoff based on ref. 41). In contrast, only 40% models for random promoter regions predicted a distance below 4.5 Å to the same promoter positions (*SI Appendix*, Fig. S6*C*).

Together with our understanding of effectostimulins, these data paint a SUGR-1-centric network of interactions that underlies the upregulation of effectors in the subventral gland cells at the very earliest times of host infection, based on host-derived effectostimulins.

### Downregulation of *sugr-1* Inhibits Host invasion.

**Fig. 5.**
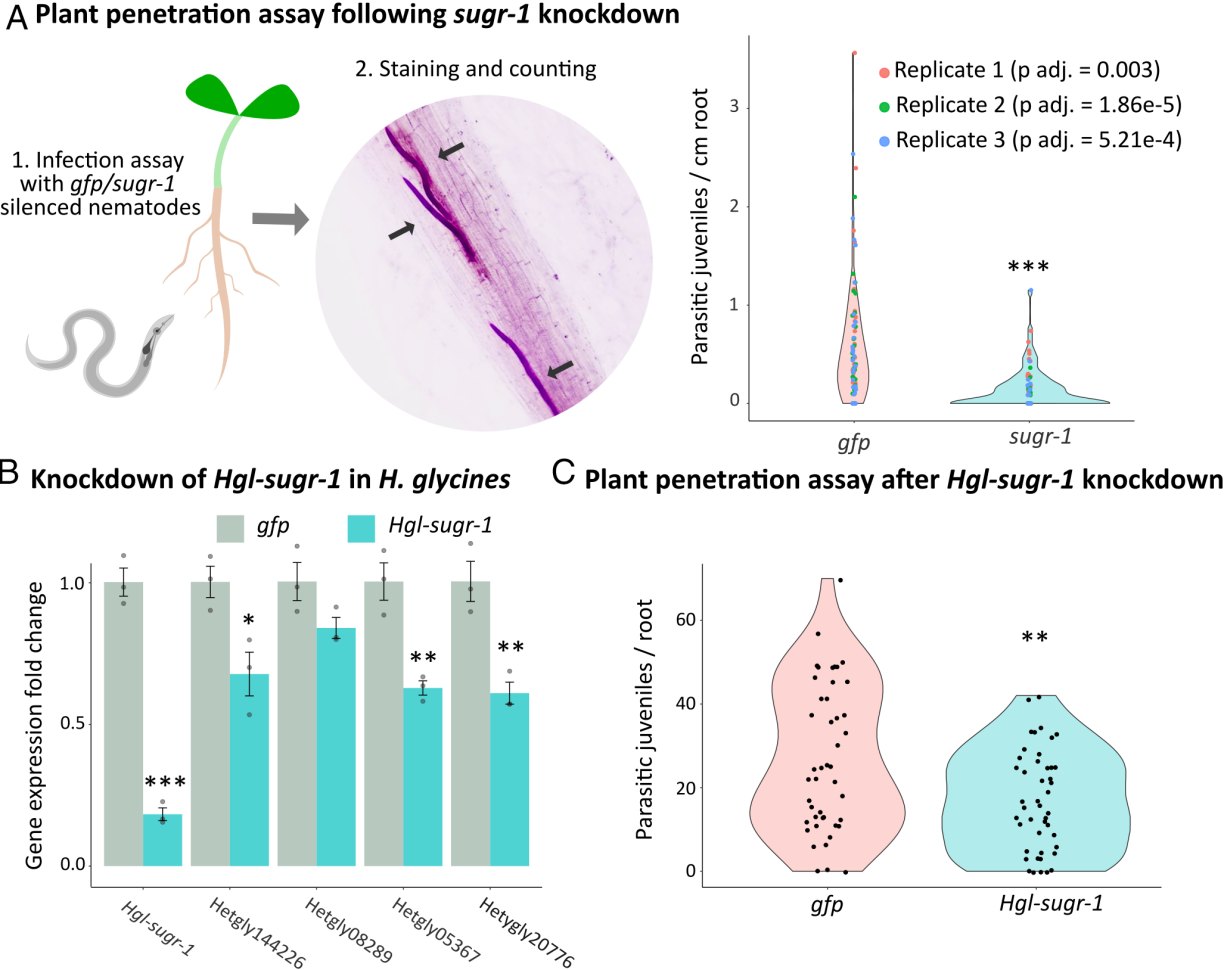
SUGR-1 is required for full pathogenicity. (*A*) Mustard plants were infected with *H. schachtii* second-stage juveniles (J2s) following RNAi-mediated silencing of *sugr-1* or *gfp* (control). The impact on nematode parasitism was determined by J2s/root. Asterisks indicate a significant difference compared to the *gfp* control at FWER < 0.001 (n = 152, Games–Howell test, colors indicate repetitions). *P*-values corresponding to comparisons within the repetitions are indicated in the plot legend. (*B*) *H. glycines sugr* (*Hgl-sugr-1*) was silenced in the same manner, and the effect on gene expression of it and four corresponding canonical subventral gland effectors was tested by qPCR and data normalized using the Pfaffl method. Asterisks indicate significantly different treatments compared to the respective *gfp* control (*P < 0.05; **P < 0.01; ***P < 0.001; two-sample *t* test two-sided; n = 3). (*C*) Plant penetration assay with *H. glycines* J2s on *Glycine max*, following RNAi-mediated silencing of *Hgl-sugr-1* or *gfp* (control). Asterisks indicate a significant difference compared to the *gfp* control at *P* < 0.01 (n = 45, Games–Howell test).

Given that SUGR-1 regulates the expression of effectors in the subventral gland, including 14 homologs of effectors involved in cell-wall degradation which have each been validated for their role in plant penetration (11, 12, 14, 16, 17, 38), we tested the phenotype of *sugr-1* knockdown by RNA interference with a penetration assay. Silencing *sugr-1* significantly reduced the number of J2s observed inside the root by over 80% compared to the *gfp*-silenced control after 10 h of infection (Fig. 5*A*, Games–Howell test, n =152, FWER < 0.001). This demonstrates the involvement of SUGR-1 in host colonization and, given that a moderate reduction of *sugr-1* expression is amplified to a much larger reduction in pathogenicity, highlights the importance of SUGR-1 signaling in general.

*H. schachtii* is both an economically important pathogen in its own right, and the model cyst nematode. Its close sister species, the soybean cyst nematode *H. glycines,* is the most economically important cyst nematode globally and the most damaging pathogen of any kind to US soy production (42). Importantly, these species have a common origin of parasitism, and remarkable conservation in effector repertoire [93% of *H. schachtii* effectors have a homolog in *H. glycines* (25)]. Given their relatedness, we were able to identify a single unambiguous homolog of SUGR-1 in *H. glycines* (Hetgly00282, 80% identical across 100% query coverage, and only three amino acids different in the DBD). Knockdown of *H. glycines sugr* (*Hgl-sugr-1*) resulted in a concomitant knockdown of three out of four canonical subventral gland effectors tested (a pectate lyase, Hetgly20776; an expansin, Hetgly05367; and a gycosyl hydrolase 53 arabino-galactanase, Hetgly14426—Fig. 5*B*). Analogous to SUGR-1, Hgl-SUGR-1 acts predominantly as a positive regulator, activating the expression of 57/86 regulated genes (*SI Appendix*, Fig. S7). Furthermore, proteins predicted to be secreted are significantly enriched (approximately 1.6 times more than expected) in Hgl-SUGR-1 activated genes (as determined in hypergeometric tests; *P*-value < 0.05), and 58% of the GO terms associated with Hgl-SUGR-1 activated genes are also found for SUGR-1 activated genes (Dataset S2). Importantly, knockdown of *Hgl-sugr-1* resulted in a significantly reduced number of *H. glycines* J2s per root (Fig. 5*C*, Games–Howell test, n = 45, *P* < 0.01). Taken together, these data suggest that SUGR-1 and Hgl-SUGR-1 regulate similar processes.

## Discussion

### Effectostimulins.

All pathogens must tailor gene expression to their environment. In the case of cyst nematodes, this is strikingly evident in light of their biology. Juvenile worms have extremely limited energy reserves: They can remain dormant in the egg for decades, hatch, locate, and enter the host, migrate through host cells, and establish a feeding site—all without feeding (43). Here, we show that *H. schachtii* effectostimulins—signals found inside plant roots—are a) able to stimulate the production of effectors and effector producing machinery (Figs. 1 *B* and *D* and 4 *A*–*C* and *SI Appendix*, Fig. S3); b) are discrete isolatable molecules (Fig. 4*C*); and c) are absent from the nonhosts tomato and rice (*SI Appendix*, Fig. S3). Thereby, effectostimulins fulfill a fundamental requirement to spare resources when the nematode must and promote parasitism when it counts.

We posit that effectostimulins must be distinct from host attraction signals because maximal effector expression before reaching the host would be prohibitively wasteful. Conceptually, an effectostimulin must signal that a permissive site within the host has been reached. Therefore, they need not necessarily be descriptive of the host per se (although they can be, *SI Appendix*, Fig. S3) but they must be conserved enough to reliably induce such profound changes in pathogen physiology, gene expression, and behavior. Indeed, reliability of the signal may partly explain why effectostimulins of the *H. schachtii:*mustard pathosystem are small in number (at least three) and redundant. These data, together with our assertions above, will inform hypotheses on the nature of the signals and the future experiments to identify them.

Why would a host genome maintain genes responsible for producing effectostimulins? The most natural parallel lies with susceptibility (S) genes: an established concept where hosts encode genes required for pathogen virulence. Importantly, and in spite of this other function, S genes are valuable targets for deriving resistant plants using genome editing (44, 45).

Our conjecture, then, is that although the molecules will almost certainly be distinct, effectostimulins are likely a generalizable concept to other, if not all, pathosystems. Viewed through this lens, existing examples from the literature (18, 46–49) support generalizability. For example, Bell et al. (18) show that effector gene expression in the migratory nematode *Pratylenchus coffeae* is activated by root exudates, while Lu et al. (48) show that infective larvae of entomopathogenic nematodes (equivalent to the J2 stage of plant-parasitic nematodes) become activated by host homogenate (ground up insects) and release effector-like lethal venom proteins. Here, the parallels go even deeper: Homologs of the upregulated genes in this example (e.g., fatty acid- and retinol-binding proteins) are also among the SUGR-1 regulated effectors. In the saprophytic fungus *Neurospora crassa* Wu et al. (49) show that parallel signaling network where the production of fungal cell wall degrading enzymes is activated by the presence of carbon sources in plant cell walls and regulated by several TFs. Further afield, the type III secretion system of phytopathogenic bacteria has been known for some time to be induced by minimal medium and plant-derived signals (50). Indeed, we are unaware of a pathosystem which would not need to tailor gene expression to its host environment.

### A Feedforward Loop for Host Entry.

The data presented allow us to build a conceptual model for effector regulation in this system. We posit that effectostimulins contained within plant cells are released upon the very earliest stages of host probing with the nematode stylet. They then activate the transcriptional regulator *sugr-1*, which orchestrates effector production (including many cell wall degrading enzymes). Effector production leads to increased cell penetration (11–19), releasing yet more effectostimulins. This model is therefore, by its nature, a feedforward loop for host entry.

We demonstrated the presence of multiple activating signals and TFs, which may perform two, not necessarily mutually exclusive, functions: First, fine-tuning of effector production based on the host/environment. Alternatively, or in addition, such redundancy may increase robustness of the system. Nevertheless, in spite of this redundancy, we demonstrate that disrupting only one component, in this case SUGR-1, is sufficient to disrupt the system.

### Future Identification of Other Regulators and Virulence Determinants.

Effectors sit at the crux of engagement between kingdoms of life. Here, we identified the first gene required for effector production in any plant-parasitic nematode. Among the SUGR-1-activated genes encoding putative secreted proteins, 46.7% are not known effectors. Understanding the regulators of effectors may therefore represent a method of effector discovery in this system. In addition, three of the top five most connected TFs to the effector network in ref. 25 are also found in the Extract up cluster, validating our approach and suggesting more regulators of effector production (other glands/times) are within reach.

For other eukaryotic pathogens, we have some understanding of both positive and negative regulators of effector production. Positive regulators include orthologs of the TF Wor1 that have been implicated in virulence of several phytopathogenic fungi (51–56), and orthologs of Pf2, first shown to regulate effectors in the fungus *Alternaria brassicicola* (57–62). Negative regulators of effector gene expression prior to plant infection include Rgs1 of *Magnaporthe oryzae* (63). Despite these intriguing insights, integration of these regulators into signaling cascades starting with activating host cues remains incomplete. A holistic model that incorporates effectostimulins could extend the potential impact yet further.

### Routes to Application.

The identification of SUGR-1 as a perturbable regulator of nematode virulence opens the door to promising routes to nematode control, targeting effector production instead of individual effectors. Disrupting the nematode internal machinery that regulates effector production is promising because: i) disrupting the production of effectors disrupts all associated effectors at the same time; and ii) the machinery involved in effector production is hidden from the plant immune system and not genetically primed for evolution, likely leading to more durable resistance (28).

The understanding of effectostimulins themselves provides opportunity. For example, modulating effectostimulin metabolism by conventional breeding, CRISPR/Cas genome editing (64), or even root microbiome engineering (65), are all in principle plausible. The finding that SUGR-1 is a nuclear hormone receptor highlights further possibilities. Nuclear hormone receptors are often bound by ligands resulting in activation, repression, or relocation and are, therefore, considered “druggable” TFs (39). This makes screening for substances that block SUGR-1 directly eminently feasible.

Cyst nematodes belong to the economically most damaging plant-parasitic nematodes, they are the dominant nematode threat in UK/northwest Europe, and the most damaging pathogen in US soybean production (42). Control measures are extremely limited and the few nematicides available are being successively removed from the market (66). The identification of SUGR-1 as a regulator of nematode virulence promises to expand our toolkit in controlling these devastating pests. Importantly, we show that disrupting SUGR-1 signaling likely disrupts similar processes across the genus, potentially extending the applicability to other important agricultural pests. Moreover, nematodes are not only capable of agricultural and ecological catastrophes, but can also parasitize humans and other animals via the secretion of effectors. This context highlights immense potential impact: disrupting effector production could, in principle, be additionally applied to the fields of human and veterinary medicine, or indeed any pathogen that secrets effectors.

## Materials and Methods

### Common Material.

*S. alba* (cv. albatross), *A. thaliana* (Columbia-0), *Solanum lycopersicum* (cv. Moneymaker), *Oryza sativa* (cv. Nipponbare*),* and *H. schachtii* populations “Bonn,” originally from Germany [as per the reference genome (31)] and “IRS,” originally from The Netherlands, were used in this study. For the yeast-one-hybrid screen, the *Saccharomyces cerevisiae* Y1HGold strain (Takarabio) was used. For bacterial transformation chemically competent cells of the *Escherichia coli* strain DH5α were used. All primers used are available in Dataset S3.

### Effectostimulin Extraction and Associated Analyses.

#### Extraction.

*S. alba* (white mustard) seeds were sterilized with 20% bleach solution (Parazone) for 20 min and grown on wet filter paper at 21 °C for 7d. Alternatively, to separate effectostimulins inside and outside roots, plants were grown in pipette tip boxes filled with 200 mL sterile, ultrapure water. To collect root diffusate, the water was exchanged after 7 d and collected 48 h later. To prepare extract, roots were ground in ultrapure water (0.5 g/1 mL), centrifuged at 10,000 rpm for 2 min and the supernatant was collected. For size exclusion, the extract was centrifuged in vivaspin columns (<3 kDa MWCO; Cytiva) at 4 °C. For ion removal (Fig. 4*B*), Pierce strong ion exchange columns (ThermoFisher) were used following the supplier’s instructions and to test heat stability the extract was boiled at 95 °C for 15 min. If needed, the extract was concentrated using a Concentrator plus (Eppendorf) at 45 °C until all liquid was removed and resuspended in the required volume. To generate data shown in Fig. 1, Extract (<3 kDa) was concentrated 3:1 to resemble biological conditions. Diffusate was used nonconcentrated (Diffusate) and concentrated 5:1 (Diffusate conc.). For all other experiments, the extract was concentrated 6:1 to achieve high activation levels.

*A. thaliana* and *S. lycopersicum* (Tomato) seeds were sterilized with 20% bleach solution (Parazone) for 20 min. *O. sativa* (Rice) seeds were briefly washed with 70% ethanol and sterilized with 3% bleach solution for 20 min. Subsequently, *A. thaliana, S. lycopersicum*, and *O. sativa* seedlings were grown on ½ MS medium (Duchefa Biochemie) at 21 °C for 10 d (7 d for *O. sativa)*. To prepare extract, 0.5 g/mL roots were ground in ultrapure water (0.1 g/mL for *A. thaliana* due to the thinner roots), centrifuged at 10,000 rpm for 2 min and the supernatant was collected. The extract was concentrated 3:1 as described previously.

#### Application to nematodes.

*H. schachtii* cysts were obtained from infected sand (Stichting IRS), isolated using sieves (4,000, 2,000, 500, 125, 63 microns), and transferred to hatching jars (Jane Maddern Cosmetic Containers). Hatching was induced by 3 mM Zinc chloride solution, jars kept at 21 °C and J2s were collected every 2 to 3 d. At least 15,000 J2s per replicate were treated with 50 µL of *S. alba* root extract, *S. alba* root extract fractions or *S. alba* root diffusate at 21 °C and 700 rpm for 4 h. As a control, 50 µL of sterile, ultrapure water was added instead. Subsequently, nematodes were flash frozen in liquid nitrogen and stored at − 80 °C.

#### RNA extraction and qPCR.

Frozen *H. schachtii* J2s were ground to powder in a Geno/Grinder 2010 (Spex Sample Prep) in three 30 s long cycles at 1,200 strokes/min. Subsequently, total RNA was extracted from each sample using the RNeasy Plant Mini Kit (Qiagen) following the manufacturer’s instructions and using both the optional QIAshredder columns and on-column DNAse digestion. RNA purity and concentration were determined using a NanoDrop One spectrophotometer (Thermo Fisher Scientific) and a Qubit RNA High Sensitivity Assay kit (Thermo Fisher Scientific). cDNA was synthesized with 400 ng RNA and Superscript iv (ThermoFisher) following the manufacturer’s instructions and using the optional RNAse H digestion and oligodT15 primers (Promega). qRT PCR was performed with the LUNA Universal qPCR Master Mix (NEB) following the manufacturer’s instructions and 1 µL of cDNA. The qPCR data were normalized using the Pfaffl method (67) against two reference genes (Hsc_gene_6993 and Hsc_gene_2491). Primers used are available in Dataset S3. Either one-way ANOVA and Tukey HSD multiple pairwise comparisons or a Kruskal–Wallis test followed by Dunn’s test were performed using R version 4.2.1. The assumptions of normality and variance homogeneity were checked by visual inspection of QQ plots with standardized residuals and residuals versus fitted plots. As an additional criterion, the Shapiro–Wilk test and Levene’s test were used. Plots were generated using the ggplot2 v3.4.2 package (68) and figures made in Inkscape v1.1. All qPCR measurements were taken on distinct samples.

#### HPLC.

HPLC analysis was performed using a Shimadzu HPLC (Shimadzu Europa GmbH) comprising Nexera X2 binary pump and autosampler with 500 µL sample loop, a Prominence column oven and diode array detector, and fraction collector. The system was controlled using Shimadzu’s Lab Solutions software (version 5.72). Separation was achieved using a YMC-Pack Pro C18 column, 250 mm × 10.0 mm ID S-5 µm 12 nm (YMC Europe GmbH Dinslaken). The column was maintained at 40 °C and a gradient used for elution at 4.0 mL/min flow rate, with initial composition of 95% mobile phase A (0.1% formic acid) and 5% mobile phase B (acetonitrile) changing to 100% B over 16 min, held isocratic at 100% B for a further four min before returning to the initial composition over 1 min and re-equilibrating the column for a further 9 min. 200 µL of sample was injected and fractions were collected every 1 min. Fractions were pooled from a total of eight sample runs and evaporated to dryness. Subsequently, fractions were resuspended in 250 µL ultrapure water.

### Effector Network Analyses.

The manually curated effector definitions from ref. 25 were used in this paper, generated using a combination of homology to previously published effectors (“knowns”), and direct gland cell sequencing (“putative”). A transcriptional network of predicted *H. schachtii* effectors was generated as in ref. 25 with an arbitrary edge threshold set at a distance correlation coefficient above 0.975. Distance correlation coefficients between the eight Extract upregulated TFs and predicted effectors were calculated and a network was generated. Of these eight TFs, six were connected to predicted effectors with a correlation coefficient of 0.975 or above. The presence or absence of each predicted effector gene or TF in the Extract upregulated dataset was added to the network as a node attribute. The number of connections with predicted effectors for each TF was added as a node attribute and used to determine the height in the z axis. The network was visualized using Gephi v0.10.1 (69). Scripts for transcriptional network analyses can be found at: https://github.com/BethMolloy/Effectorome_H_schachtii/tree/main (70).

### Characterization of SUGR-1.

Hsc_gene_14352 was identified as the most highly connected of the 8 candidates’ TFs in the Extract up cluster (Fig. 1*D*), and indeed the second-most highly connected TF of any kind, to the effector network (25). Therefore, it was further characterized. Domain prediction of SUGR-1 (Hsc_gene_14352) was performed using InterPro (71). Protein structure was predicted using AlphaFold (72) and visualized using CCP4MG (73). The SUGR-1 gene model was created using the R package genemodel v1.1.0 (74).

### RNA Sequencing and Analyses.

RNA sequencing and library construction were performed by Novogene. The mRNA library was prepared by poly-A enrichment (poly-T oligo-attached magnetic beads), fragmentation, cDNA synthesis (using random hexamer primers), followed by end-repair, A-tailing, adapter ligation, size selection, amplification, and purification. Illumina sequencing was performed using 150 bp paired-end reads, generating 5G raw data per sample. RNA sequencing reads are available under ENA accession PRJEB71637 (75). All reads were analyzed with FastQC v0.11.9 (76) and 10 bp were trimmed using BBduk in BBTools v38.18 (77). Reads were mapped to the reference *H. schachtii* genome (31) using STAR v2.7.9a (78) and counted using HTseq v0.13.5. (79). Differentially expressed genes were identified in R version 4.2.1 (80) using the DESeq2 v1.38.3 package (81) following pairwise comparison of all samples (|log2FC| ≥ 0.5 and *P*adj ≤ 0.001). Hierarchical clustering was performed after scaling using the hclust() function of the stats v4.2.1 package. Volcano plots were plotted using EnhancedVolcano v1.16.0 (82). GO term enrichment analyses were performed using the gprofiler2 v0.2.1 package (83). Gene set enrichment was determined by hypergeometric enrichment tests. For the *sugr-1* silencing experiment, the same packages were used but with FastQC v0.11.8, BBduk v38.34, STAR v2.7.0e, HTSeq v0.12.4, R v3.5.2, DESeq2 v1.22.2, EnhancedVolcano v1.0.1, and gprofiler2 v0.1.6.

### In Situ Hybridizations.

The multiplexed HCR in situ was performed as described in ref. 37. The probes to the designated genes (Hsc_gene_14352; Hsc_gene_2726; Hsc_gene_21727 *eng2*) and in situ reagents were designed and purchased from Molecular Instruments, Inc. The images were acquired on a Leica Stellaris 8 FALCON confocal microscope with minor adjustments made to the brightness and contrast. 3D projections were created with the Leica Cyclone 3DR software. The images were prepared using ImageJ (84). No further image manipulation was performed.

In situ hybridizations were performed using ppJ2 of *H. schachtii* following previously published methodology (85). Specific primers were designed to amplify a product for each of the candidate effector genes using a cDNA library produced from ppJ2s (Dataset S3). The resulting PCR products were then used as a template for generation of sense and antisense DIG-labeled probes using a DIG-nucleotide labeling kit (Roche, Indianapolis, IN). Hybridized probes within the nematode tissues were detected using an anti-DIG antibody conjugated to alkaline phosphatase and its substrate. Nematode segments were observed using a DP73 digital Olympus camera mounted on a Bx51 Olympus microscope.

### Yeast One Hybrid.

#### Plasmid construction.

Promoters of SUGR-1-activated genes (up to 2 kb upstream intergenic DNA) were amplified from *H. schachtii* gDNA [Q5 polymerase (NEB) according to the manufacturer’s instructions] and cloned into the SacI (NEB) digested pAbAi plasmid (Takarabio). All promoters were additionally analyzed in two parts [proximal (b) and distal (a) halves]. For this, one promoter half was cut out of the plasmid by mutagenesis PCR [PrimeSTAR Max polymerase (Takarabio) following the manufacturer’s instructions]. *sugr-1, safta,* and *satfb* were amplified from *H. schachtii* cDNA [Q5 polymerase (NEB)] and cloned into the PCR-amplified pDEST22 plasmid (Invitrogen). Cloning was performed using the In-Fusion HD cloning master mix (Takarabio) following the supplier’s instructions. Bacterial transformation was performed using the heat shock method (30 min ice, 35 s 42 °C, 5 min ice) and plasmids were extracted using the Monarch Plasmid Miniprep kit (NEB) following the manufacturer’s instructions.

#### Generation of promoter bait:TF prey yeast strains.

Bait yeast strains were generated by transforming the *S. cerevisiae* Y1HGold strain (Takarabio) with the promoter (bait) plasmids. Prior to transformation the bait plasmids were linearized in the *URA3* gene by PCR or restriction digest [BbsI/Esp3I/BstBI (NEB)] to allow integration into the genome. Subsequently, the bait strains were transformed with a TF (prey) plasmid to generate bait:prey strains. As a control, all bait strains were also transformed with the pDEST22 empty vector. For yeast transformation, yeast overnight cultures (1.5 mL grown SD-ura or YPDA medium at 28 °C) were pelleted and resuspended in 10 µL TE-LiAc solution (10 mM Tris-HCl pH 8.0, 1 mM EDTA, 0.1 M Lithium acetate), 10 µL salmon sperm DNA (Invitrogen), 300 ng plasmid DNA, and 500 µL PEG-TE-LiAc solution (40% PEG 3500, 10 mM Tris-HCl pH 8.0, 1 mM EDTA, 0.1 M Lithium acetate). After shaking at 200 rpm/30 °C for 30 min followed by 45 min heat shock at 42 °C, yeast were washed with sterile ultrapure water and grown on selective SD medium for 3 d at 28 °C.

#### Yeast-one-hybrid screens.

The generated bait:prey yeast strains were grown overnight in liquid SD -ura/trp medium at 28 °C and 250 rpm and subsequently diluted in water to OD600 = 0.6. Finally, 2 µL drops of yeast suspension were plated in five 1:5 serial dilutions on 100 mm square plates (Thermo Fisher) with selective SD medium and increasing concentrations (0.07 to 7 µg/mL) of the antibiotic Aureobasidin A (Takarabio). Yeast were grown for 4 d at 28 °C and images taken on days 2 to 4 using a GBox gel doc system (Syngene). Due to the different background of native TF:bait binding, pictures shown represent the Aureobasidin A concentration and time point at which no or limited growth was observed for the empty vector control yeast strain. Only interactions observed in at least three technical replicates are shown. Pictures were cropped and figures were made using Inkscape but all comparisons shown stem from the same plate. Original pictures are available at DRYAD accession https://doi.org/10.5061/dryad.vmcvdnd0q (86).

### SUG Box Identification.

Proximal 5′ promoter regions of all *H. schachtii* genes were predicted using a series of custom python scripts (https://github.com/sebastianevda/H.schachtii_promoter_regions) (87). In brief, proximal 5′ promoter regions were defined as n bases of intergenic space, where available, upstream of the coding start site. In this study, 800 bp was used. From this database of promoter regions, subsets were extracted and compared. Comparisons included SvG effectors vs DG effectors; and SvG effectors, J2 to 10hpi expressed genes, or SUGR-1-regulated effectors vs a random set of 666 genes. Genes used for these comparisons are listed in Dataset S4. Enriched motifs were identified using HOMER (88).

SUGR-1:DNA complexes were predicted using the AlphaFold3 (89) server for 15 promoter regions containing a perfect match to CTGAACAA[A|T] and 10 bp upstream and downstream DNA as well as 15 random 29 bp promoter sequences of the random set of 666 genes. Furthermore, for a motif-containing region in the Hsc_gene_21726 promoter single motif bases were mutated (C↔A or T↔G). AlphaFold3 models were visualized using CCP4MG (73). All promoter sequences used for AlphaFold3 models are listed in Dataset S3. Distances in angstrom were calculated using a custom python script https://github.com/Olaf2K/AF3_PDI_distance_Calc (90). In brief, the shortest distance between a defined region on the DNA (e.g., six adjacent bases) and a defined region on the polypeptide (e.g., five adjacent amino acids) is selected from an all vs all comparison of distances between positions in those defined regions.

### RNA Interference.

A silencing mix was prepared using 3 μg/μL dsRNA (either silencing *sugr-1* or *gfp*; ordered from Genolution, Dataset S3); 50 mM octopamine [to aid uptake (91)] and M9 buffer. *H. schachtii* J2s were soaked in the silencing mix for 48 h at 700 rpm on a thermoblock set at 21 °C. If needed, silenced J2s were subsequently flash frozen in liquid nitrogen and RNA extraction and sequencing were performed as described in the previous sections.

### Plant Penetration Assay.

Five-day-old *S. alba* plants (grown on Daishin agar at 21 °C) were inoculated with ~100 *H. schachtii* J2s (100 µL solution with 1 J2/µL; J2s were counted under a stereomicroscope) and kept in the dark for 10 h. To generate data in Fig. 5*A* nematodes were silenced in either *gfp* or *sugr-1* as described previously. For staining, roots were treated with 1% bleach for 2 min followed by treatment with boiling acid fuchsin solution for 2 min. Subsequently, roots were covered in acidified glycerol and left to destain. Nematodes were counted under a dissecting microscope. The results in Fig. 5*A* were validated in two independent experiments. Data shown represent three repeats with about 50 plates each. Statistical analysis was performed by a Games–Howell test (92, 93). Adjusted p-value corresponds to the Family Error Wise Rate (94). All measurements were taken on distinct samples.

### *H. glycines* SUGR Homology.

The *H. glycines* SUGR-1 homolog was identified using BLAST [wormbase-parasite (95)], and sequence similarity was compared to SUGR-1 using amino acid alignments in MUSCLE (96). Sense and antisense RNA were synthesized in a single in vitro reaction using the MEGAscript® RNAi Kit (Thermo Fisher Scientific, Waltham, MA) according to the manufacturer’s instructions, with an incubation period of 6 h to enhance RNA yield. The resulting dsRNA product underwent purification (97), integrity examination through 1.2% agarose gel electrophoresis. Approximately 30,000 freshly hatched *H. glycines* J2s (TN10) per biological replicate were soaked in a mixed buffer containing 3 μg/μL dsRNA in 1/4 M9 buffer (43.6 mM Na_2_HPO_4_, 22 mM KH_2_PO_4_, 2.1 mM NaCl, 4.7 mMNH_4_Cl), 1 mM spermidine [to aid efficiency (98)], and 50 mM octopamine (91) at 26 °C on a rotator covered with aluminum foil to maintain a dark environment. After 24 h of incubation, J2s were washed three times with Nemawash (5 μL of Tween20 in 50 mL of MES buffered water) before being flash frozen in liquid nitrogen if needed.

RNA extraction was performed using the Nucleospin microRNA kit (Macherey-Nagel, Hoerdt, France) following the manufacturer’s instructions. The isolated RNA was then reverse transcribed into first-strand cDNA using LunaScript RT SuperMix (NEB) according to the manufacturer’s instructions. Real-time PCRs were conducted using iTaq universal SYBR Green super mix (Bio-Rad) on a CFX96 Real-time PCR Machine (Bio-Rad Laboratories, Inc., Hercules, CA) following the manufacturer’s instructions. Thermocycler conditions comprised an initial denaturation cycle at 95 °C for 30 s, followed by 40 cycles at 95 °C for 5 s and 58 °C for 30 s, concluding with amplicon dissociation. The experimental design included three biological replicates and three technical replicates. Expression levels of *Hgl-sugr-1* and four subventral gland effectors (Hetgly05367, Hetgly08289, Hetgly20776, and *Hetgly14426*) that are homologs of SUGR-1 regulated effectors, identified by BLAST, were normalized to the endogenous *HgGAPDH* (*CA939315.1*) using the Pfaffl method (67). Results were confirmed with a second reference gene (*HgActin*). Statistical analysis was performed by two-sample t-tests using R v4.2.1. Plots were generated using the ggplot2 v3.4.2 package and figures made in Inkscape v1.1. RNA sequencing was performed (by Novogene) and analyzed as described in previous sections.

*Glycine max* seeds (Williams 82) were surface-sterilized with 70% ethanol for 2 min and then with 50% bleach for 10 min, followed by three rinses in sterile water. Sterilized seeds were placed on wet filter paper with MES buffer inside a Petri plate and incubated in a growth chamber at 26 °C. 5 d old seedlings of the same length (1 cm) were used for the experiments. A 23% Pluronic F-127 (PF-127) (Sigma-Aldrich) gel was prepared as per (99). SCN infection was assessed in a 6-well tissue culture plate. Three milliliters of Pluronic gel were poured into each well, and seedlings were placed in each well at 15 to 20 °C. After the gel solidified, approximately 100 J2s/50 μL of *H. glycines* were inoculated at the root tip of each seedling using a pipette tip. Nine plates were included in the experiment for each treatment. Three biological replicates were used for each treatment (*gfp* and *Hgl-sugr1*), with each biological replicate consisting of 15 technical replicates (15 individual seedlings). In total, 45 plants for *gfp* and 45 plants for *Hgl-sugr1* were included in the analysis. After 24 h, plants were harvested from the gel by briefly placing the plates over an ice bath. Due to the slight decrease in temperature, the gel liquefied, allowing the plantlets to be easily extracted without damaging the root system. Roots were stained with acid fuchsin following the method outlined by ref. 100, and the number of J2s penetrating the root was counted using a stereomicroscope. Photographs were taken. All measurements were taken on distinct samples.

## Supplementary Material

Appendix 01 (PDF)

Dataset S01 (XLSX)

Dataset S02 (XLSX)

Dataset S03 (XLSX)

Dataset S04 (XLSX)

## Data Availability

Raw reads deposited in ENA Accession PRJEB71637 (75). Scripts unique to this manuscript are deposited under the following GitHub accessions: https://github.com/sebastianevda/H.schachtii_promoter_regions (87) and https://github.com/Olaf2K/AF3_PDI_distance_Calc (90). Network files are deposited under DRYAD Accession https://doi.org/10.5061/dryad.vmcvdnd0q (86). Plasmids generated are available upon request.
