# Supplementary material for: The SUbventral-Gland Regulator (SUGR-1) of nematode virulence
Source: Proc Natl Acad Sci U S A. 2025 Mar 10;122(11):e2415861122. doi: 10.1073/pnas.2415861122 (PMC11929438; doi:10.1073/pnas.2415861122)
Supplement: Supplementary file 1 — Appendix 01 (PDF) [file pnas.2415861122.sapp.pdf]

PNAS

Supporting Information for

The SUbventral-Gland Regulator (SUGR-1) of nematode virulence

Authors:

Clement Pellegrin[1,6+], Anika Damm[1,6], Alexis L. Sperling[1], Beth Molloy[1], Dio S. Shin[1], Jonathan Long[1], Paul Brett[2], Tochukwu Chisom Iguh[1], Olaf P. Kranse[1], Andrea Díaz-Tendero Bravo[1], Sarah Jane Lynch[1], Beatrice Senatori[1], Paulo Vieira[3], Joffrey Mejias[4], Anil Kumar[4], Rick E. Masonbrink[5], Tom R. Maier[4], Thomas J. Baum[4], Sebastian Eves-van den Akker[1*]

Affiliations:

[1] The Crop Science Centre, Department of Plant Sciences, University of Cambridge, Cambridge CB2 3EA, UK.

[2] Department of Biochemistry and Metabolism, John Innes Centre, Norwich NR4 7UH, UK.

[3] Mycology and Nematology Genetic Diversity and Biology Laboratory, United States Department of Agriculture - Agricultural Research Service, Beltsville, Maryland, 20705, USA

[4] Department of Plant Pathology, Entomology and Microbiology, Iowa State University, 2213 Pammel Dr., Ames, IA 50011, USA

[5] Genome Informatics Facility, Iowa State University, 448 Bessey Hall, Ames, IA 50011, USA

[6]Contributed equally

[+]Current address: European Commission, Joint Research Centre (JRC), B-2440 Geel, Belgium

*Corresponding Author: Sebastian Eves-van den Akker (se389@cam.ac.uk)

This PDF file includes:

Figures S1 to S7
Legends for Datasets S1 to S4
SI References

Other supporting materials for this manuscript include the following:

Dataset S1 to S4

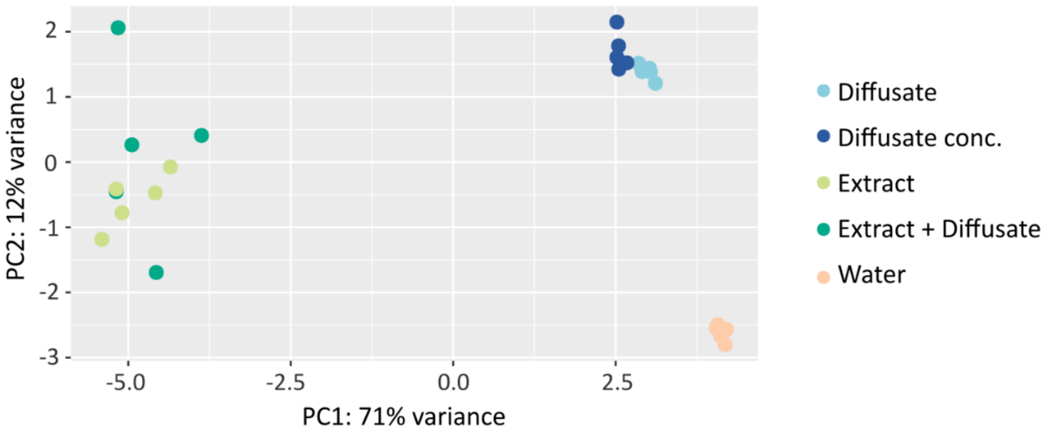

Figure S1. Principal Component (PC) Analysis of RNA sequencing data shown in Figure 1B.

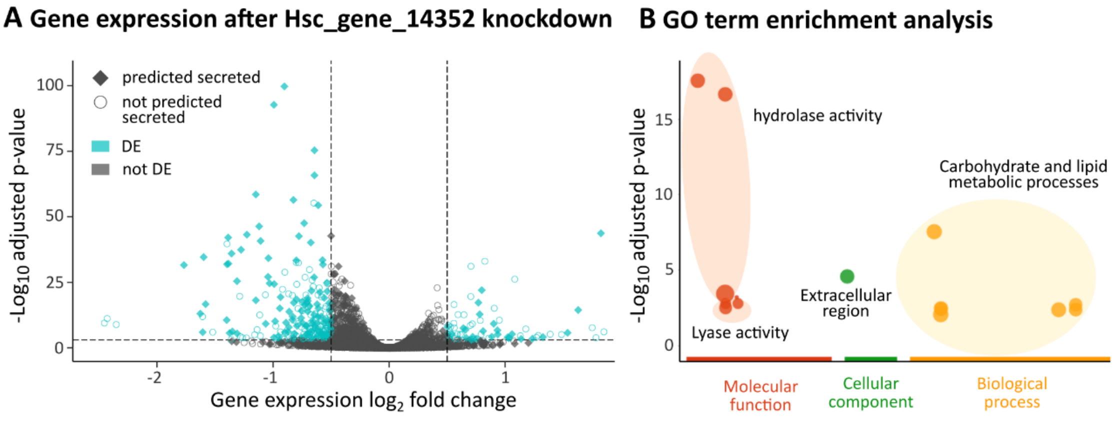

Figure S2. A) *Heterodera schachtii* gene expression following Hsc_gene_14352 knockdown vs. *gfp* control. Differentially expressed genes (n=3; |log2FC|≥0.5 & padj≤0.001) are highlighted in cyan. Genes encoding proteins predicted to be secreted are diamond shapes. **B)** GO term enrichment analysis of Hsc_gene_14352 activated genes ($p<0.05$).

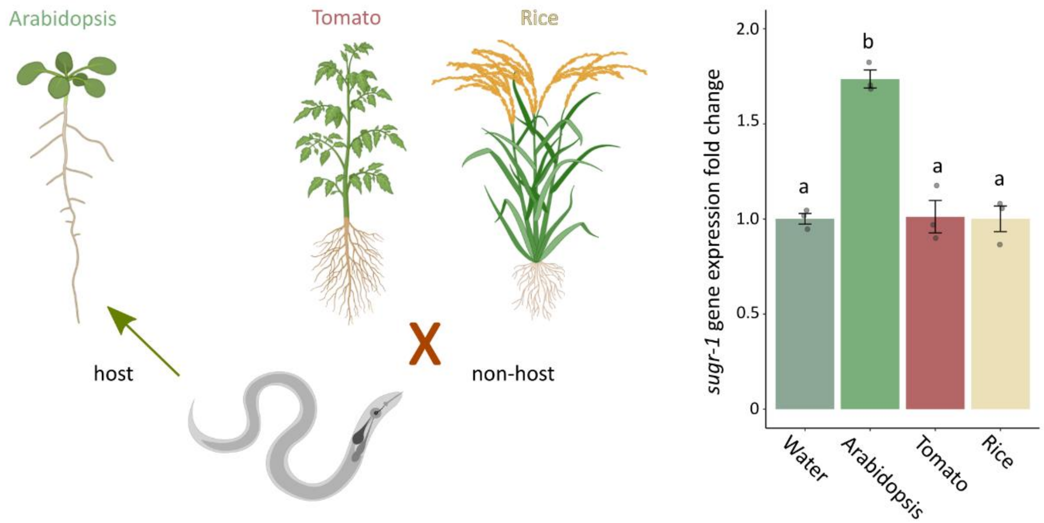

Figure S3. Effectostimulins in hosts and non-hosts. *sugr-1* gene expression following treatment of *H. schachtii* J2s with root extract of the host plant *Arabidopsis thaliana* and the non host plants tomato (*Solanum lycopersicum)* and rice (*Oryza sativa)*. *sugr-1* gene expression was determined by qPCR and data normalised using the Pfaffl method. Treatments with the same letter are not statistically significantly different at $p<0.05$ (Tukey HSD). Drawings of plants created in BioRender.

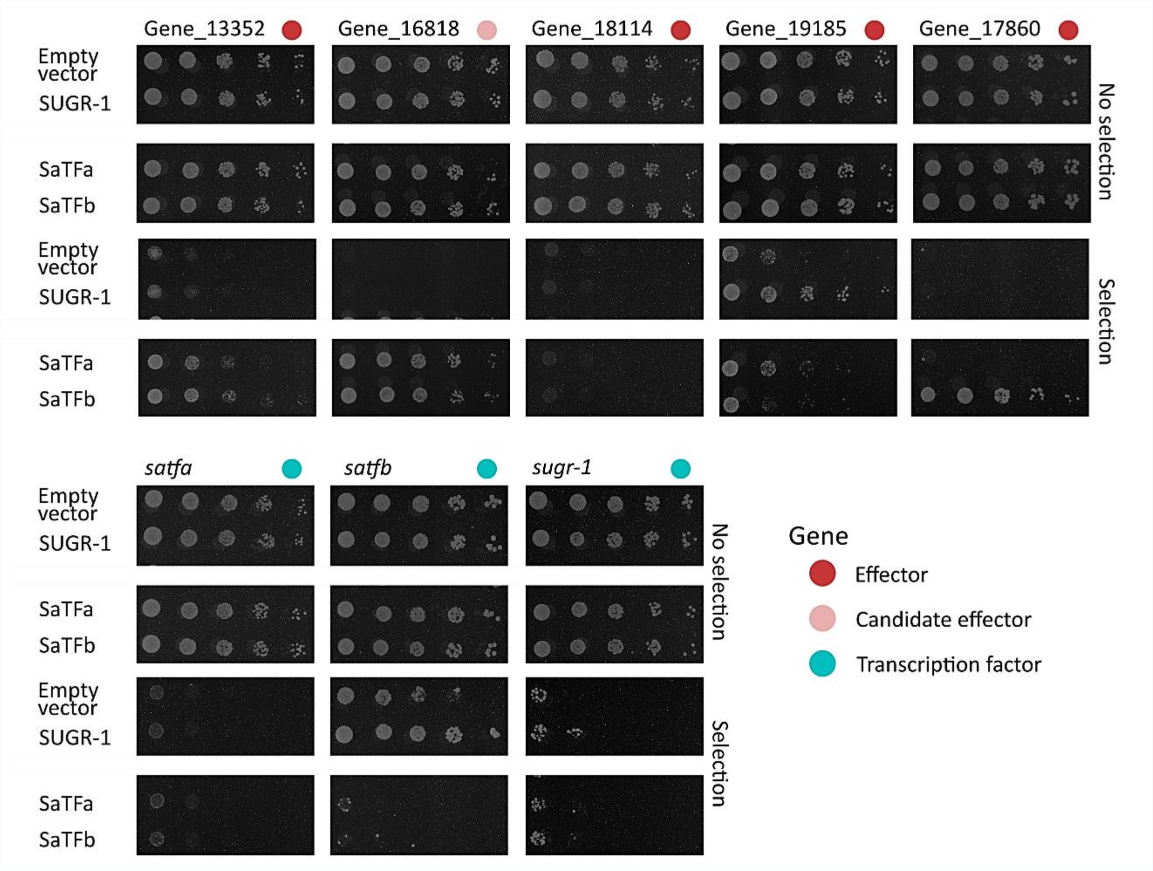

Figure S4: Yeast-one-hybrid pictures for full length promoters. Binding of SUGR-1, SaTFa (SUGR activated Transcription Factor a), and SaTFb (SUGR activated Transcription Factor b) to promoters (up to 2kb upstream intergenic DNA) of SUGR-1-activated genes (Figure 3B). Promoters of effector genes (as predicted in (1) and/or validated via *in situ* hybridisation (Figure 3C) are highlighted with red circles. *Saccharomyces cerevisiae* growth with or without Aureobasidin A selection was compared to the pDEST22 empty vector control in a 1:5 serial dilution. Pictures were cropped but all comparisons for the same promoter come from the same plate.

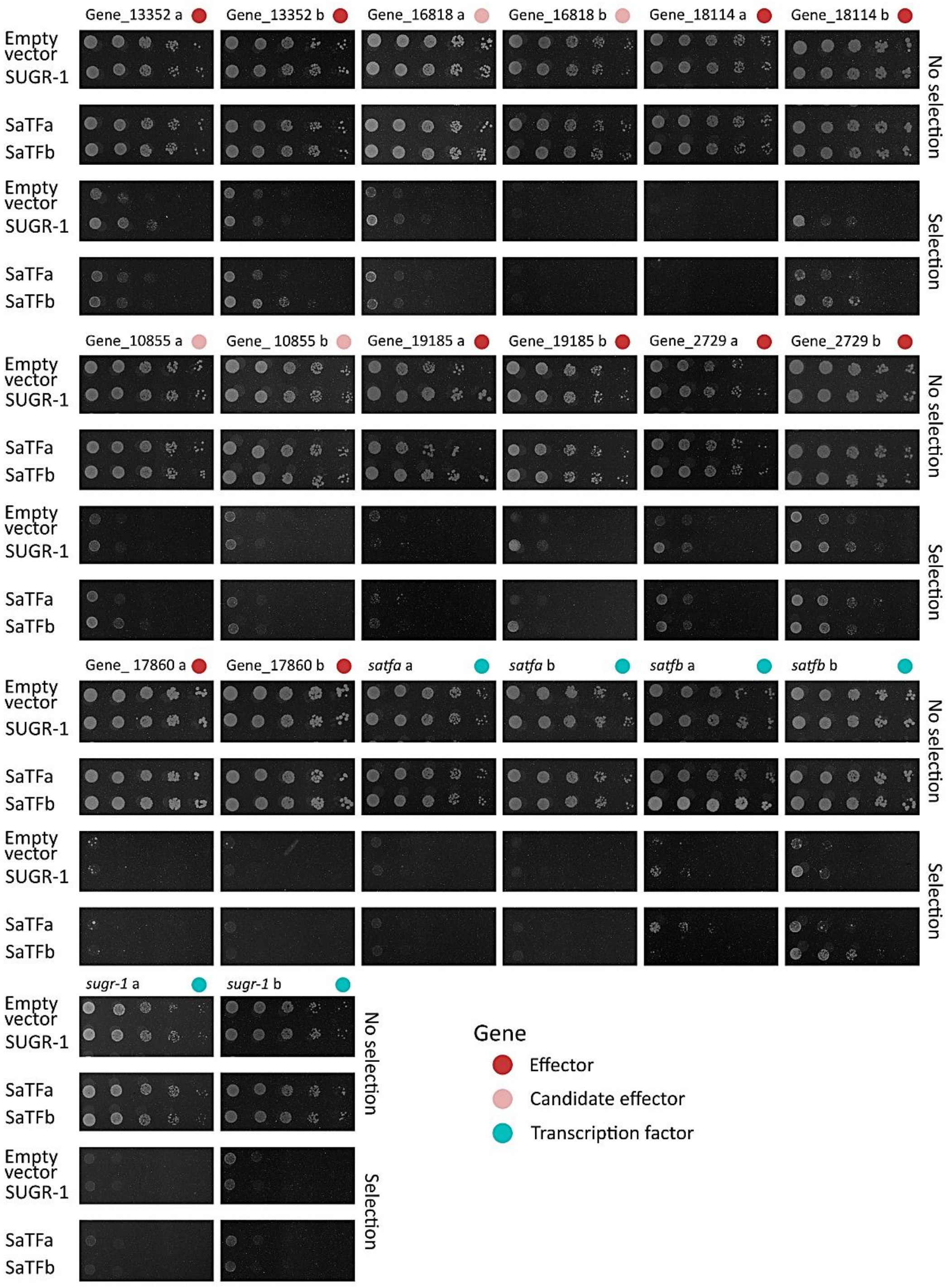

Figure S5: Yeast-one-hybrid pictures for promoter halves. Binding of SUGR-1, SaTFa, and SaTFb to promoter halves of SUGR-1-activated genes (Figure 3B) which were additionally analysed in two parts (proximal (b) and distal (a) halves). Promoters of effector genes (as predicted in (1) and/or validated via *in situ* hybridisation (Figure 3C) are highlighted with red circles. *S. cerevisiae* growth with or without Aureobasidin A selection was compared to the pDEST22 empty vector control in a 1:5 serial dilution. Pictures were cropped but all comparisons for the same promoter come from the same plate.

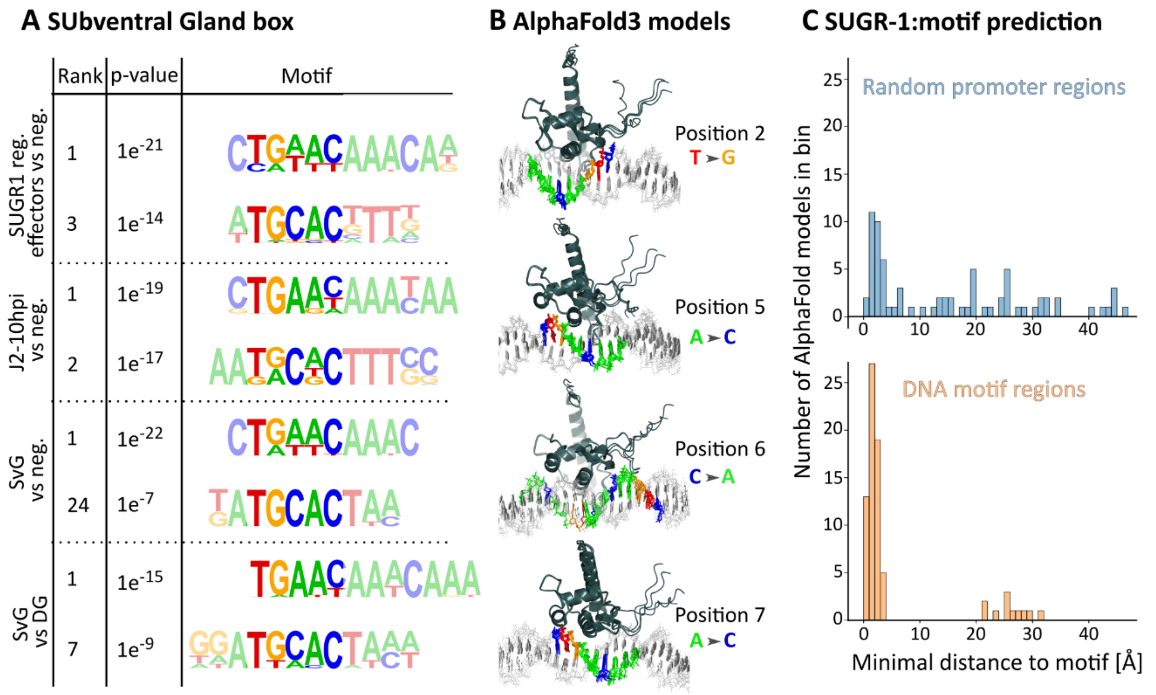

Figure S6: The SUG box. A) DNA motifs enriched in promoters of subventral gland effectors (SvG), SUGR-1 regulated effectors, or effectors found in the J2-10 hours post infection (hpi) supercluster from (2). Motif enrichment analyses with respective promoters (800bp from start codon) were compared to a negative control set (neg.) or dorsal gland effectors (DG). Gene names of comparisons are listed in supplementary Dataset S4. **B)** SUGR-1:DNA complex formation was predicted with a 29bp Hsc_gene_21726 promoter region containing the SUG box version CTGAACAAA mutated in positions 2, 5, 6, and 7. All five models predicted for the same DNA sequence are shown superposed. **C)** Minimal Distance of the SUGR-1 C-terminal loop to the motif in 29bp promoter regions containing CTGAACAA[A|T] (orange) vs minimal distance to the same position in random 29bp promoter regions (blue).

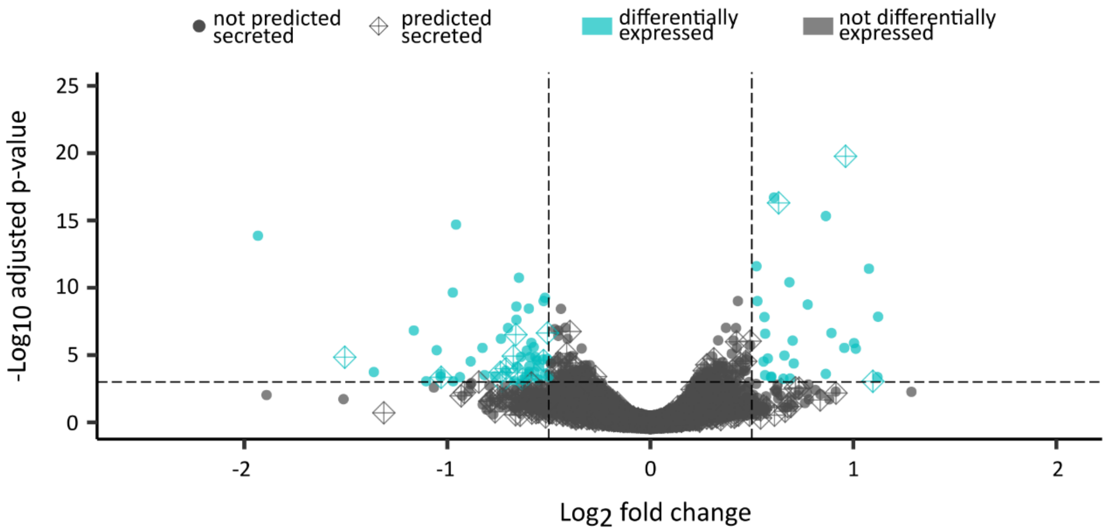

Figure S7: *Heterodera glycines* gene expression following *Hgl-sugr-1* knockdown vs. *gfp* control. Differentially expressed genes (n=3; |log2FC|≥0.5 & padj≤0.001) are highlighted in cyan. Genes encoding proteins predicted to be secreted are diamond shapes.

Legends for Dataset S1 to S4

Dataset S1: GO term enrichment analysis of Hsc_gene_14352 activated genes (p<0.05). GO enrichment was determined using the R package gprofiler2.

Dataset S2: GO term enrichment analysis of *Hgl-sugr-1* activated genes (p<0.05). GO enrichment was determined using the R package gprofiler2.

Dataset S3: Primers and constructs used to generate the data presented in this paper.

Dataset S4: Gene sets and command used for differential motif discovery with HOMER.

SI References

1. B. Molloy, *et al.*, The origin, deployment, and evolution of a plant-parasitic nematode effectorome. *PLoS Pathog.* **20**, e1012395 (2024).

2. S. Siddique, *et al.*, The genome and lifestage-specific transcriptomes of a plant-parasitic nematode and its host reveal susceptibility genes involved in trans-kingdom synthesis of vitamin B5. *Nat. Commun.* **13**, 6190 (2022).
